# Treatment of a flunixin meglumine overdose with intravenous administration of lipid emulsion and therapeutic plasma exchange in a Nigerian dwarf buck kid (*Capra aegagrus hircus*)

**DOI:** 10.1111/jvim.16124

**Published:** 2021-05-02

**Authors:** Emmanuelle Marie Butty, Caroline Ann McKinney, Amanda Jane Prisk

**Affiliations:** ^1^ Department of Clinical Sciences, Small Animal Internal Medicine Cummings School of Veterinary Medicine at Tufts University North Grafton Massachusetts USA; ^2^ Department of Clinical Sciences, Large Animal Internal Medicine Cummings School of Veterinary Medicine at Tufts University North Grafton Massachusetts USA; ^3^ Department of Clinical Sciences, Large Animal Surgery Cummings School of Veterinary Medicine at Tufts University North Grafton Massachusetts USA

**Keywords:** flunixin meglumine, goat, intravenous lipid emulsion, NSAID, therapeutic plasma exchange, toxicity

## Abstract

A 12 week‐old Nigerian dwarf (*Capra aegagrus hircus*) buck kid was hospitalized for management of obstructive urolithiasis. Postoperatively, he was inadvertently administered 16‐times greater than his calculated dose of a nonsteroidal anti‐inflammatory drug (NSAID; 17.5 mg/kg flunixin meglumine, IV). The goat was treated with intravenous administration of lipid emulsion (ILE) prior to membrane‐based therapeutic plasma exchange (mTPE) under general anesthesia. The increased coagulability inherent to small ruminants in comparison with dogs and cats warranted specific adjustments in the prescription of anticoagulation, blood flow, and filtration fraction to avoid circuit clotting during mTPE. Serum flunixin meglumine concentration measured before, during, and after mTPE revealed marked reduction in drug concentration. After the combined treatments, no clinical evidence of NSAID gastrointestinal or renal toxicosis was detected. This case report describes successful management of flunixin meglumine overdose in a small ruminant using combined ILE and mTPE.

**Abbreviations**
ACT: activated clotting time
cTPE: centrifuge‐based therapeutic plasma exchange
HES: hydroxyethyl starch
ILE: intravenous administration of lipid emulsion
mTPE: membrane‐based therapeutic plasma exchange
NSAID: nonsteroidal anti‐inflammatory drug
Qb: blood flow
RCA: regional citrate anticoagulation
TPE: therapeutic plasma exchange
TS: total solid
UFH: unfractionated heparin

## 1 INTRODUCTION

Flunixin meglumine is a commonly prescribed nonsteroidal anti‐inflammatory drug (NSAID) that acts through its inhibition of cyclooxygenase and the synthesis of prostanoids. Flunixin has a small molecular weight of 296.24 g/mol but is highly protein bound in goat serum (84.8%) with a volume of distribution of 0.35 L/kg and clearance of 110 mL/h/kg in adult, female dairy goats.^1^ The elimination half‐life after intravenous injection is 3.6 hours.^2^ Adverse effects might occur secondary to prolonged or excessive dosing and include renal injury, gastrointestinal ulceration, diarrhea, anorexia, and central nervous system impacts.^3^, ^4^ To the authors' knowledge, singular dose ranges associated with the acute development of toxicosis in specific body systems has not been established for goats. For large animal species, management of flunixin toxicosis relies upon intravenous fluid diuresis and administration of gastroprotectant medications and prostaglandin analogues. In small animal medicine and occasionally, smaller‐sized large animals, intravenous administration of lipid emulsion (ILE) has become an adjunct therapy in NSAID toxicosis^5^, ^6^, ^7^, ^8^ with increasing use of extracorporeal blood purification strategies.^9^, ^10^, ^11^, ^12^

In addition to being a component of parenteral nutrition, ILE is used in both human and veterinary medicine as an antidote in toxicoses associated with the overdose of lipophilic compounds, such as tricyclic antidepressants, calcium‐channel blockers, β‐blockers, macrocyclic lactone anthelmintics, and, more recently, NSAIDs.^5^, ^6^, ^7^, ^8^, ^13^ While the mechanism of action has not been definitively elucidated, the most commonly accepted proposed mechanism is providing a “lipid sink” within the intravascular space, with the lipophilic compounds then sequestered in the newly‐formed lipid compartment.^5^

Therapeutic plasma exchange (TPE) is an extracorporeal blood purification technique. The basic principle relies on removal of circulating pathogenic substances from the plasma. Separation of plasma from whole blood can be achieved by either centrifuge‐based (cTPE) or membrane‐based TPE (mTPE). In cTPE, the centripetal force from rotation of a blood chamber is used to separate cellular and acellular blood components based on differences in density. In mTPE, a nonselective membrane filters solutes based on size, allowing proteins to convectively cross a capillary membrane and leaving behind the blood cellular components.^14^

As the literature stands, several case reports^9^, ^10^, ^11^ and 1 retrospective study of NSAID intoxication treated with mTPE have been published for dogs.^12^

## 2 CASE DESCRIPTION

### 2.1 Initial case information

A 12‐week‐old, 11.4 kg Nigerian dwarf buck (*Capra aegagrus hircus*) kid was admitted for a 48‐hour history of stranguria and lethargy. After diagnosis of obstructive urolithiasis, surgical intervention via tube cystotomy using previously‐described standard technique was pursued.^15^ Perioperatively, the goat received antimicrobials (ceftiofur [Naxcel; Zoetis, Kalamazoo, Michigan] 4.4 mg/kg IV q12h), NSAID (flunixin meglumine [Covetrus North America, Dublin, Ohio] 1.1 mg/kg IV q12h), crystalloid fluids (Plasmalyte A [Baxter Healthcare Corporation, Deerfield, Illinois] 120 mL/kg/day), gastroprotectant (pantoprazole [Auromedics Pharma LLC, East Windsor, New Jersey] 1 mg/kg IV q24h), and thiamine supplementation (Henry Schein Animal Health, Dublin, Ohio) 30 mg/kg SQ q24h).

Approximately 96 hours postoperatively, the goat received an inadvertent overdose of NSAID (17.5 mg/kg flunixin meglumine IV), with the error recognized within several minutes of administration. Due to the magnitude of overdose, ILE therapy followed by mTPE was pursued. Approximately 30 minutes after the adverse event, ILE therapy was initiated (Intralipid 20%; Baxter Healthcare Corporation, Deerfield, Illinois) with a 1.5 mL/kg bolus followed by a 0.25 mL/kg/minute constant rate infusion over 30 minutes.

#### 2.1.1 Membrane based therapeutic plasma exchange

The goat was sedated with butorphanol (0.2 mg/kg IV) and midazolam (0.25 mg/kg IV). General anesthesia was induced with ketamine (2.5 mg/kg IV) and midazolam (0.2 mg/kg IV). Anesthesia was maintained with 1.5% to 2% sevoflurane. Electrocardiography, indirect blood pressure by oscillometry, pulse oximetry, end‐tidal CO2, and temperature were monitored. The oral and oropharyngeal areas were regularly suctioned throughout the procedure.

The anesthetized goat was placed in left lateral recumbency and the area of the right external jugular vein was aseptically prepared. A 11.5 F X 20 CM double lumen dialysis catheter (straight duo‐flow catheter, Medcomp, Harleysville, Pennsylvania) was placed in the right jugular vein and the correct placement in the right atrium was confirmed with 1 lateral radiograph.

The mTPE was performed on a Prismaflex platform (Gambro Lundia AB, Lund, Sweden) using a TPE 2000 circuit with a priming volume of 125 mL (Baxter Healthcare Corporation, Deerfield, Illinois). The circuit was primed with 0.9% NaCl with 5000 U/L of unfractionated heparin (UFH) (Heparin Sodium; Sagent Pharmaceuticals, Schaumburg, Illinois). The goat was estimated to be euhydrated and weighed 11.1 kg. Packed cell volume and total solids (TS) were 27% and 6.2 g/dL. The blood and plasma volumes were estimated to be 888 mL (8% of the body weight) and 648 mL (73% of the blood volume). Because of the large amount of NSAID administered, the goal of the mTPE prescription was to provide 1.5 plasma volume exchanges which should decrease the concentration of a completely protein‐bound drug by 78%.^16^ The replacement fluid consisted of a 3% HES solution by diluting—in a 1 : 1 ratio—a 6% hydroxyethyl starch (HES) (Vetstarch; Zoetis, Kalamazoo, Michigan) with NaCl 0.9%. This formulation of replacement fluid has been successfully used in dogs.^9^, ^10^, ^11^, ^12^, ^17^ Fresh plasma was collected from donor goats in the institutional herd and represented the last 250 mL of the replacement fluid. Unfractionated heparin was used for systemic anticoagulation throughout mTPE and was monitored with activated clotting time (ACT) measurements every 15 minutes (ACT Plus Automated Coagulation Timer System, Medtronic, Minneapolis, Minnesota). The goal was to maintain the ACT at twice the baseline to avoid circuit clotting.

The session was initiated 4 hours after the overdose had occurred. The goat's ACT prior to the mTPE was 99 seconds (reference interval: 91 ± 3 seconds) and served as a baseline measurement.^18^ A first bolus of 50 U/kg of UFH was administered IV after the first ACT, 30 minutes prior to the beginning of the session. As the ACT was still 97 seconds, the goat received a second bolus of 50 U/kg of UFH which increased the ACT to 178 seconds at the beginning of the session. This is at least double the amount of UFH required than the typical initial amount for a dog.^12^ The goat was also started on a CRI of UFH at 50 U/kg/h. Because of the species‐related coagulation tendencies confirmed by the important amount of heparin required to increase the ACT, risk of clotting was considered high.^19^ The heparin CRI was increased to 100 U/kg/h after 20 minutes because of a constant rise in the transmembrane pressure suspected to be secondary to clotting within the filter capillaries. The filtration fraction was then kept low between 12% and 17% and the blood was flowing at a median rate of 155 mL/min (range, 75‐165). The initial ionized calcium was low‐normal (1.12 mmol/L, range, 1.12‐1.4). A calcium gluconate (Calcium Gluconate Injection USP 10%; Fresenius Kabi, Lake Zurich, IL) CRI was started at 1 mL/kg/h at the beginning of the session and increased to 1.5 mL/kg/h when the replacement fluid was switched to fresh plasma to prevent a clinically important decline in calcium related to the citrate from the plasma administration. The mTPE session took 1 hour and 23 minutes to exchange a total plasma volume of 972 mL. The median ACT was 204 seconds (range, 154‐238). The median ionized calcium level remained stable at 1.17 mmol/L (range, 1.12‐1.3). The ACT, PCV, and TS at the end of the session were 204 seconds, 23% and 4.4 g/dL, respectively.

**FIGURE 1:**
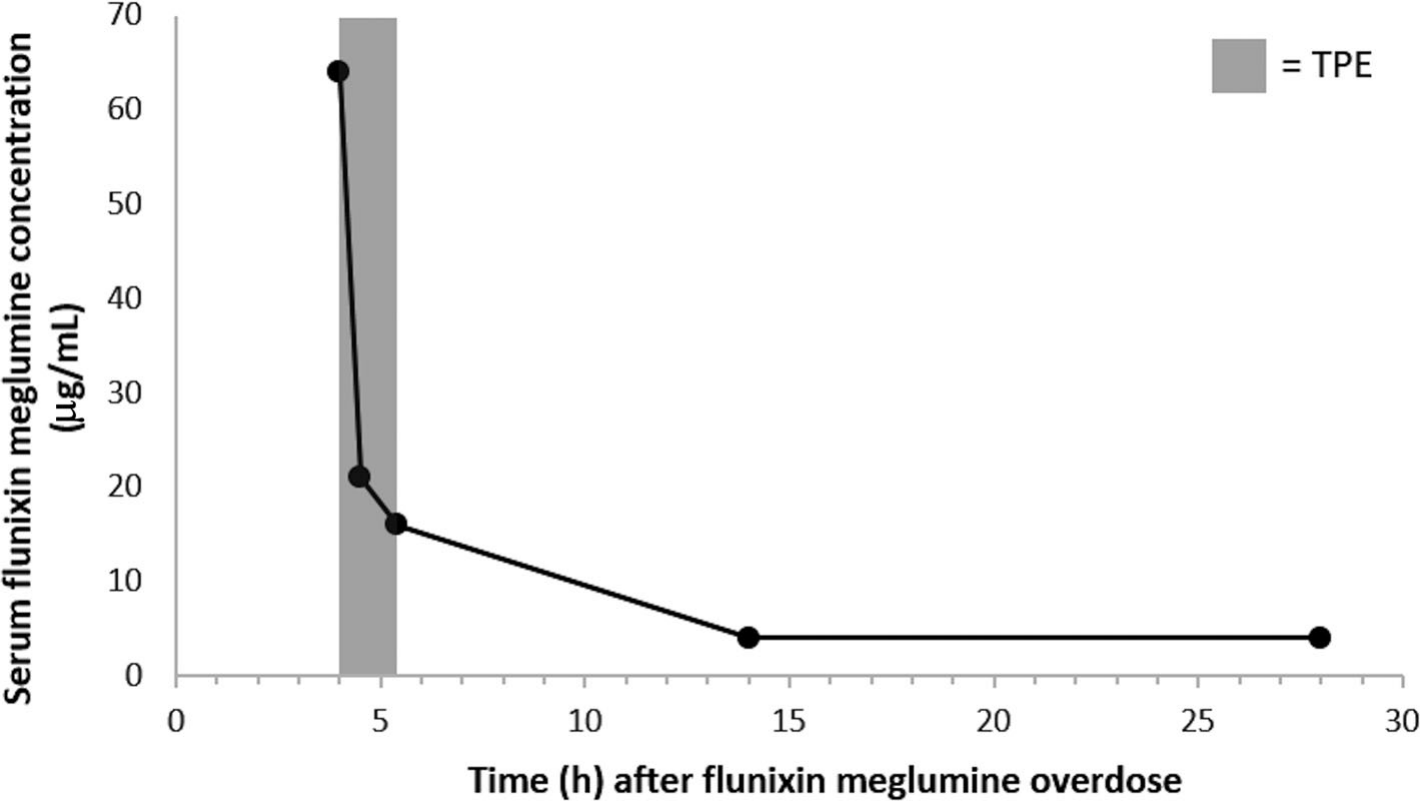
Serum flunixin meglumine concentrations after the overdose (*T* = 0) measured at the initiation of membrane‐based therapeutic plasma exchange (mTPE) (*T* = 4), during mTPE (*T* = 4.5), at the conclusion of mTPE (*T* = 5.4), 10 hours after mTPE (*T* = 14), and 24 hours after mTPE (*T* = 28). Marked reduction of the drug concentrations by 75% during the treatment time and continued but slower reduction of drug concentrations up to 93.75% of the initial value 10 hours after cessation of mTPE, with no further reduction 24 hours after mTPE

After the overdose, blood samples for measurement of flunixin concentrations were collected at the initiation, during, and at the conclusion of mTPE, as well as 10 and 24 hours after mTPE. Serum samples were frozen and stored at −80°C for future analysis via liquid chromatography‐mass spectrometry (Texas Veterinary Medical Diagnostic Laboratory at Texas A&M University, College Station, Texas) (Figure 1).

### 2.2 Management after mTPE

In recovery, the goat had a subdued mentation, tachycardia, and tachypnea prompting the administration of additional pain medications (butorphanol 0.1 mg/kg IV [Torbugesic; Zoetis, Kalamazoo, Michigan]; transdermal fentanyl patch 25 mcg/h [Apotex Corporation, Weston, Florida]) and intranasal oxygen supplementation. Thoracic radiographs identified bilateral interstitial opacity in ventral and, less severely, caudodorsal lung fields. The goat was given 1 mg/kg furosemide IV (Salix; Intervet Inc, Madison, New Jersey), to address potential pulmonary edema, which mildly improved his respiratory effort and rate and his caudodorsal interstitial pattern, though the ventral opacity remained, consistent with aspiration pneumonia. The goat also developed a fever (104.4 F) and leukocytosis (27 K/μL, range, 4‐13; neutrophils 17.78 K/μL, range, 1.2‐7.2), prompting altered antimicrobial treatment (florfenicol [NuFlor Merck, Madison, New Jersey] 20 mg/kg IM q 24 hours). Gradually, the goat's vital signs, radiographs, and blood‐work normalized (WBC 14.2 K/μL), allowing removal of the nasal cannula used to deliver oxygen therapy. Regular measurements of blood chemistries did not reveal evidence of azotemia (creatinine: 0.4‐0.8 mg/dL; BUN 10‐20 mg/dL) nor any other abnormalities apart from electrolyte derangements including low‐normal calcium (iCa 1.14 mmol/L, range, 1.12‐1.4), hypokalemia (2.3 mmol/L, range, 3.5‐4.5) and hypophosphatemia (2.8 mg/dL, range, 4‐8). Electrolytes were initially supplemented by intravenous administration and then transitioned to oral supplementation (DiCal; AniMed, Winchester, Kentucky). Diuresis was induced with an increased rate of isotonic fluid therapy (120 mL/kg/day) for the first 24 hours after mTPE, then titrated to the goat's hydration status prior to gradual discontinuation. Regular urine specific gravity measurements were appropriate throughout recovery (1.026‐1.045 after discontinuation of fluid therapy). The goat also received gastroprotectants (pantoprazole 1 mg/kg IV q 24 h; sucralfate 15 mg/kg PO q8h [Teva Pharmaceuticals USA Inc, North Wales, Pennsylvania]), a prostaglandin E2 analogue (misoprostol [Lupin Pharmaceutical Inc, Baltimore, Maryland] 4 mcg/kg PO q 12 hours), and vitamin supplementation (thiamine 40 mg/kg SQ q 12 hours; Vitamin E [Elevate; Kentucky Performance Products, Versailles, Kentucky] 20 IU/kg PO q 24 hours). The goat remained on phenazopyridine (Westminster Pharmaceuticals LLC, Tampa, Florida) 4 mg/kg PO q12h for urinary pain relief and ammonium chloride (AHC Products, Inc, Winchester, Kentucky) 450 mg/kg PO q 24 hours to acidify the urine in light of the calculi's magnesium ammonium phosphate‐like appearance appreciated at surgical removal (complete analysis declined). His Foley catheter remained unclamped during the initial recovery. The physical examination, complete blood count and chemistry were normal on follow‐up evaluation with his referring veterinarian 7 days after discharge. The catheter was removed 25 days later after confirmation of urethral patency.

## 3 DISCUSSION

This case report describes successful management of flunixin meglumine overdose in a small ruminant using combined ILE and mTPE.

As has been reported in a human patient with multiple drug toxicoses,^20^ ILE was administered prior to mTPE in this patient due to the magnitude of overdose, with this concurrent treatment strategy shown to improve survival in a rabbit model of clomipramine toxicosis.^21^ While no data exist in veterinary medicine regarding the advantage of combining ILE with mTPE, ILE has the advantage of being easily and rapidly administered. Since NSAIDs have a short half‐life and are lipophilic, compartmentalization of the drug occurs into the temporary lipid phase storage depot created by the ILE. In theory, this results in a higher concentration of drug in the plasma with less free drug available to the tissues, therefore decreasing its toxic effects.^5^ This might also have kept the majority of the drug in the intravascular space while awaiting mTPE. While typically well‐tolerated in both human and veterinary medicine, potential adverse effects of ILE include acute anaphylactoid reactions, which occurs in <1% of human patients, and fat overload syndrome subacutely, often attributed to administration of excessive volumes or a high rate of administration.^5^ No such adverse effects were appreciated in the case reported.

The use of mTPE in this goat had pharmacological and clinical justifications. Despite its small size, flunixin is highly protein bound and thus cannot be dialyzed. Clinically, no antidote is available and the overdose of 16 times beyond the intended dose placed the goat at risk of gastrointestinal, renal and potential central nervous system injury.^3^, ^4^ TPE could accelerate the clearance of this drug since theoretically, blood concentrations would have returned to therapeutic concentrations after 14 hours (4 half‐lives). Thus, the benefits of this treatment modality were exceeding the risk of potential complications described with mTPE.^12^, ^17^

Comparing mTPE for goats against that of dogs, 3 major differences were identified:

At the outset, acquiring fresh plasma from goats would likely be an impediment to the procedure, which is not presented for mTPE in dogs. For our goat, the return of fresh plasma was made possible by collection from goats in the institutional herd.

Second, goats are generally not companion animals with a cooperative disposition. General anesthesia was needed during the entire session to ensure adequate blood flow through avoidance of catheter complications commonly associated with agitation. It is important to note that even though the oral and oropharyngeal areas were regularly suctioned throughout the procedure, the pneumonia that developed after the procedure was suspected to be aspiration of gastric content related to the anesthesia.

Finally and most importantly, the goat requires an anticoagulation prescription unique to the species‐related coagulation tendencies and careful monitoring thereof to avoid clotting complications in the extracorporeal system.

The pattern of hypercoagulability in this case mirrors previous reports of small ruminants having increased coagulability, measured by thromboelastography, in comparison to humans and other species.^22^ In previous work evaluating caprine response to heparin bolus and constant‐rate‐infusion, goats required higher doses than sheep and humans to reach anticoagulation thresholds (ACT >400 seconds).^19^ Other anticoagulation strategies such as regional citrate anticoagulation (RCA) limited to the blood in the extracorporeal circuit could have been attempted to overcome the goat's hypercoagulability. However, a safe and efficient RCA protocol is currently only developed in dogs in veterinary medicine.^23^

Due to the concern of clotting in the extracorporeal circuit, the blood flow and filtration fraction were also adjusted:

The blood flow (Qb) started at 75 mL/min was increased to 115 mL/min and 150 mL/min after 15 and 30 minutes, respectively. The goals were to decrease the time the blood was spent circulating in the machine and to decrease the filtration fraction to avoid the risk of clotting by hemoconcentration within the filter capillaries. Since the transmembrane pressure was constantly rising, this filtration fraction was decreased from 16% to 12% after 40 minutes of treatment. The transmembrane pressure was decreased and stabilized for the rest of the session. The treatment resolved successfully without interruption or requiring a change of the circuit. After the session, the filter showed a mild to moderate amount of clotting macroscopically.

## CONFLICT OF INTEREST DECLARATION

Authors declare no conflict of interest.

## OFF‐LABEL ANTIMICROBIAL DECLARATION

Florfenicol (NuFlor Merck, Madison, New Jersey) was administered at the recommended dosage and via the recommended route for cattle (20 mg/kg) though at an increased interval of every 24 hours. This medication is not labeled for use in small ruminants including goats though was selected for its improved antimicrobial spectrum.

Ceftiofur sodium (Naxcel; Zoetis, Kalamazoo, Michigan) was given at an increased dosage and an alternative route from label instructions for goats (4.4 mg/kg IV q12 for 4 days).

## INSTITUTIONAL ANIMAL CARE AND USE COMMITTEE (IACUC) OR OTHER APPROVAL DECLARATION

Authors declare no IACUC or other approval was needed.

## HUMAN ETHICS APPROVAL DECLARATION

Authors declare human ethics approval was not needed for this study.
